# Supplementary material for: Selective interoception impairments and treatment effects in borderline personality disorder
Source: Transl Psychiatry. 2026 Sep 10;16:463. doi: 10.1038/s41398-026-04414-7 (PMC13562582; doi:10.1038/s41398-026-04414-7)
Supplement: Supplementary file 1 [file 41398_2026_4414_MOESM1_ESM.pdf]

Supplementary Information
Selective interoception impairments and treatment effects in Borderline Personality Disorder

Supplementary Methods

Power-analysis

We used G*Power 3 (1) to conduct an a-priori power analysis for the study based on the effect size reported in a Cochrane Review on psychological therapies for patients with borderline personality disorder (BPD) (2). The authors observed an overall medium effect size of *d* = -0.6 for dialectical behavior therapy (DBT) treatment effects on borderline symptom severity. A different study (3) found the same effect size (*d* = -0.6) for positive effects of DBT treatment on interpersonal problems in BPD. Because the study focuses on the effects of one DBT module (four weeks treatment), we assumed a reduced effect size of *d* = 0.5. To replicate the abovementioned DBT treatment effect on borderline symptom severity and interpersonal problems in BPD (with α = 0.05 and power = 0.9, one-tailed paired-sample t-test) at least 36 patients have to be tested. Assuming a possible drop-out rate of 20-25% due to the longitudinal study design, we planned to test at least 50 patients.

A previous study (4) observed large effect sizes for differences in social functioning between BPD patients and HC. Assuming a large effect (*d* = 0.8) and considering an allocation ratio of 2:1, based on the assumption of a 50% treatment response rate in the patient group, (with α = 0.05 and power = 0.9, two-tailed two-sample t-test), at least 25 HC have to be tested. To account for possible drop-outs of 10-15%, we planned to test at least 30 HC.

Residential DBT program

The residential DBT at the Karl-Jaspers-Klinik includes the following treatment components:

- Individual therapy (2 hours per week)
- Group skills training (2 hours per week)
- Group psychoeducation (1 hour per week)

- Peer group meetings (2 hours per week)
- Mindfulness group (1 hour per week)
- Individual body-oriented therapy (1.5 hours per week)
- Therapist team consultation meetings (2 hours per week)

The psychoeducation sessions offer insight into Marsha Linehan's biosocial theory of BPD, complemented by the latest theoretical developments and research findings on BPD. The mindfulness group builds upon the standard DBT skills training mindfulness module, with a stronger focus on sitting practice compared to traditional outpatient DBT. Meanwhile, the body-oriented therapy consists of education on psychomotor interactions and exercises aimed at enhancing body awareness and perception (3).

The residential DBT program at the Karl-Jaspers-Klinik is led by trained psychologists and is supported by medical doctors and nurses. It has been officially certified by the DBT Dachverband (DBT e.V.) since 2011. The staff of the DBT ward follows the German interactive skills manual for borderline patients by Martin Bohus and Martina Wolf-Arehult (5). This manual is based on the DBT skills training by Marsha Linehan (6). The content has been revised and expanded in cooperation with Marsha Linehan. It includes essential background information and instructions for conducting group skills training, as well as concrete worksheets and informational materials. In addition to the treatment sessions mentioned above, BPD patients begin their day with a morning round, where they discuss their plans and goals for the day. They end each day by reviewing their diary card with the nurses, which tracks daily events and tension curves throughout the day. Furthermore, at the Karl-Jaspers-Klinik, patients participate in additional therapeutic activities which are an integrated part of the DBT and vary depending on individual needs:

- Ergotherapy
- Dance and theater therapy
- Woodworking workshops

Patients engage in these treatments approximately about six times per DBT module. Additionally, patients meet once a week with the chief senior physician to discuss medication adjustments and with their supervising psychiatric nurse to talk about any problems during daily living.

DBT at the Karl-Jaspers-Klinik comprises three modules, each followed by a break of varying duration. Patients were recruited from all three DBT modules (1st module n=23, 2nd module n=10. 3rd module n=4).

Participants

General exclusion criteria were age under 18 or over 65 years, MRI contraindication, scars on a predefined area of 20 cm of their shins, acute suicidality, any lifetime psychotic disorders, current substance dependence, a history of traumatic brain injuries, or other neurological illnesses. The presence of a mental disorder or current or past psychiatric inpatient treatment resulted in exclusion from study participation in the HC group.

Axis I disorders were assessed by the Structured Clinical Interview for DSM-5 Disorders: clinician version (SCID-5-CV) (7). HC additionally underwent the Zanarini Rating Scale for Borderline Personality Disorders (ZAN-BPD) (8) and completed the Assessment of DSM-IV Personality Disorders (ADP-IV) (9) to confirm the absence of any current mental disorders. The average time between the last study appointment and admission to DBT was 4.15 ± 5.10 weeks. Accordingly, the HC group was assessed with a waiting period of five to nine week between pre- and post-measurement.

Psychological and clinical assessments

The Beck Depression Inventory-II (BDI-II) (10) and the Liebowitz Social Anxiety Scale (LSAS) (11) were used to measure depressive symptoms and social anxiety, respectively. Inner tension was assessed on a visual analog scale (VAS) from 0 (not at all agitated) to 100 (very agitated) approximately half an hour before participants entered the MRI scanner. Furthermore, hunger and the urge to urinate were evaluated verbally immediately before the visceral interoceptive awareness (VIA) task on a scale from 0 (no hunger/urge to urinate) to 100 (very strong) while the participant was inside the MRI. Except for the Childhood Trauma Questionnaire (CTQ), all participants completed the questionnaires before and after four weeks of residential DBT/waiting interval. Depressive symptoms and social anxiety were only analyzed at baseline to characterize the sample.

Heartbeat discrimination task

To minimize noise disturbances, the experimenter left the room and closed the door and windows. Participants were instructed not to manipulate their heart rate (e.g., by holding their breath) or measure their pulse manually. The experiment required the detection of at least three consecutive, artifact-free heartbeats to initiate a tone sequence. The task comprised 80 trials, with 40 trials featuring asynchronous tones (randomly delayed by 550 ± 75 ms) and 40 trials featuring synchronous tones (randomly delayed by 250 ± 75 ms). Task duration varied based on participants' reaction time but

generally ranged from 20 to 25 minutes. The heartbeat discrimination task was applied before and after one module (i.e., four weeks) of the residential DBT program.

The heartbeat discrimination task is a well-established paradigm originally introduced by Brener and colleagues (12) and later adapted in multiple studies, including Garfinkel and colleagues (13). Our task design, including the delay intervals for the synchronous (R-wave + 250±75 ms) and asynchronous conditions (R-wave + 550±75ms) as well as the cueing procedure, was closely based on the implementation described by Garfinkel and colleagues (13).

Auditory tones (440 Hz, 100 ms duration) were presented in relation to participants' actual cardiac cycle. In the synchronous condition, tones were generated at the beginning of the rising edge of the pulse pressure wave. In the asynchronous condition, tones were presented with an additional delay of 300 ms relative to this point, accounting for the average delay (~250 ms) between the ECG R-wave and the arrival of the pulse pressure wave at the finger (13).

The delay of around 250 ms after the R-peak in the synchronous condition was chosen because prior research has shown that, although some variability exists across individuals, tones presented within this interval are most frequently perceived as synchronous with the heartbeat. This temporal window (approximately 100-300 ms after the R-wave) also coincides with peak aortic and left-ventricular pressure (12).

To prevent participants from adapting to a fixed delay and to account for inter-individual variability in perceived synchrony, the delay of tones was randomized, following a normal distribution either around 250 ± 75 ms for the synchronous condition or around 550 ± 75 ms for the asynchronous conditions. Selecting a delay of around 550 ± 75 ms for the asynchronous condition ensured that the tone presentation occurred outside the typical temporal window in which tones are perceived as synchronous with the heartbeat.

Importantly, in both conditions tones were presented at the same overall rate (i.e., either aligned or shifted to the heartbeat). Consequently, participants could not use the rhythm of the tones or prior knowledge of their heart rate to guide their responses (13). The additional randomization of tone delays further reduced the likelihood that participants could adjust to the timings of the tone.

Visceral interoceptive awareness (VIA) task

To optimize trial arrangements (order of conditions and duration of interstimulus intervals (ISIs), which were randomized between 2.5 and 12.5 seconds), the easy-optimize-x implementation for MATLAB

(The MathWorks, Natick, MA) by Bob Spunt (https://www.bobspunt.com/easy-optimize-x/) was used. Optimal designs were generated and randomly assigned to participants, with variations between pre- and post-measurement. MRI data was acquired using a 3 Tesla Siemens MAGNETOM Prisma Scanner (Siemens AG, Erlangen, Germany) with a Siemens 64-channel head coil. For fMRI data, a T2*-weighted echoplanar (EPI) multiband sequence with a multiband acceleration factor of four was used to measure neural responses to interoceptive attention (Repetition time (TR) = 850 ms, Echo Time (TE) = 30 ms, matrix size: 76 x 76, voxel size: 2.5 x 2.5 x 2.5 mm^3, slice thickness = 2.5 mm, distance factor = 0 %, field of view (FoV) = 192 x 192 mm^2, flip angle 62°, 48 slices). To control for inhomogeneities of the magnetic field, a fieldmap was obtained prior to the VIA task and was included during preprocessing of the fMRI data (TR = 533 ms, TE (1) = 5.19, TE (2) = 7.38, matrix size: 64 x 64, voxel size: 3 x 3 x 3 mm^3, slice thickness = 3.0 mm, distance factor = 33 %, FoV = 192 x 192 mm^2, flip angle 60°, 35 slices). High-resolution T1-weighted structural images were collected at the same scanner (TR = 2000 ms, TE = 2.07 ms, matrix size: 320 x 320, voxel size: 0.8 x 0.8 x 0.8 mm^3, slice thickness = 0.75 mm, FoV = 240 x 240 mm^2, flip angle = 9°, 224 slices) and were included during the analysis of fMRI data. The fMRI VIA task was performed before and after one module (i.e., four weeks) of the residential DBT program.

Behavioral data analysis

The statistical analyses were conducted using R (14) and MATLAB release R2021a (The MathWorks, Natick, MA).

To assess possible group effects on confidence ratings of the heartbeat discrimination task, mixed-design Analyses of Covariance (ANCOVAs) including the between-subject factor group (HC, BPD), the within-subject factor trial type (incorrect, correct; e.g. the synchronous tones were recognized as synchronous in correct trials) and covariates were computed. To assess treatment-related effects, the within-subject factor time (pre, post) and the interaction of group and time were additionally included.

A mediation analysis with childhood maltreatment (CTQ scores) as predictor, self-reported interoceptive attention (MAIA-2 scores) as mediator, and interpersonal problems (IIP scores) as an outcome was conducted to examine the relationship between adverse childhood environments, resulting psychosocial deficits, and interoception as an underlying mechanism.

To measure the effect sizes, we employed Cohen's d, partial eta squared (η_p^2), and generalized η^2 (η_G^2) (15, 16). To determine the magnitude of the effects, the following commonly used benchmarks can be applied (15): for Cohen's d, a small effect is indicated by d = 0.2, a medium effect by d = 0.5, and a large effect by d = 0.8. Similarly, for both η_p^2 and η_G^2, the benchmarks are as follows: a small effect corresponds to η_p^2 = 0.01, a medium effect to η_p^2 = 0.06, and a large effect to η_p^2= 0.14.

fMRI data analysis

fMRI data were preprocessed using the standardized pipeline *fMRIPrep* 20.2.1 (17), which is based on *Nipype* 1.5.1 (18, 19). The first three volumes of each functional time series were discarded to allow for T1 equilibration. The pipeline included correction for magnetic field inhomogeneities and removal of movement components using Independent Component Analysis - Automatic Removal of Motion Artifacts (ICA-AROMA). Additionally, we checked whether the standardized derivative of the root mean squared variance over voxels (std. DVARS) of our participants exceeded a threshold of 1.5. This was the case for zero participants. Physiological noise was corrected using the Component Based Noise Correction Method (CompCor). The fMRI analysis was conducted using SPM12 (https://www.fil.ion.ucl.ac.uk/spm/), implemented in MATLAB release R2021a (The MathWorks, Natick, MA). The analysis involved a two-level approach, analyzing the data on single-subject and group level. General Linear Models (GLMs) were used, and GLM parameters were estimated using the restricted maximum likelihood method. The FAST model was applied to correct for autocorrelations. Hemodynamic responses to all three conditions (heart, stomach, target) were modeled as boxcar functions with condition duration as length. The duration of ratings, the break between runs, three cerebrospinal fluid (cfs) and two white matter (wm) CompCor components explaining the most variance in the data and cosine regressors as a high-pass filter were included as nuisance regressors.

The fMRI analysis in SPM12 involved a two-level approach based on the General Linear Model. During the first-level analysis, the heart and stomach conditions were each contrasted separately with the exteroceptive control condition. Additionally, a combined contrast was computed in which both interoceptive conditions were equally weighted to test for greater activation during interoception relative to exteroceptive control. Treatment effects were investigated by calculating differences between pre- and post-measurement data in BPD patients and HC on the first level. Second-level statistical inference included two-sample and one-sample t-tests. To define the regions of interest (ROIs), we used the Human Brainnetome Atlas (20) and merged them into a single ROI mask using the BRAinNetome Toolkit (BRANT) (21) to adjust for multiple ROIs. The individual brain regions consist of the following subregions: vmPFC (41 OrG_L_6_1, 42 OrG_R_6_1, 45 OrG_L_6_3, 46 OrG_R_6_3, 47 OrG_L_6_4, 48 OrG_R_6_4, 49 OrG_L_6_5, 50 OrG_R_6_5), amygdala (211 Amyg_L_2_1, 212 Amyg_R_2_1, 213 Amyg_L_2_2, 214 Amyg_R_2_2), dACC (179 CG_L_7_3, 180 CG_R_7_3, 183 CG_L_7_5, 184 CG_R_7_5) and the insular cortex (163 INS_L_6_1, 164 INS_R_6_1, 165 INS_L_6_2, 166 INS_R_6_2, 167 INS_L_6_3, 168 INS_R_6_3, 169 INS_L_6_4, 170 INS_R_6_4, 171 INS_L_6_5, 172 INS_R_6_5, 173 INS_L_6_6, 174 INS_R_6_6).

To validate that the fMRI VIA task elicited activation in an interoception-related brain network, we analyzed data of HC at baseline using the ROI mask comprising the insular cortex, dACC, amygdala, and vmPFC with a one-sample t-test on the contrast interoception>exteroception.

fMRI representational similarity analyses (RSAs)

All ROI-based RSAs were computed using The Decoding Toolbox (TDT) (22), and bootstrapped t-tests were calculated with 10,000 replicates.

Missing values

Five BPD patients have missing data on years of education, and two BPD patients did not complete the MAIA-2 for the pre-measurement. For one BPD patient, we imputed the covariates hunger and urge to urinate using the mean of the group for the fMRI VIA task at baseline. Due to technical problems, baseline assessments of the heartbeat discrimination task were available only for 49 BPD patients and 26 HC, resulting in longitudinal data from 26 BPD patients and 26 HC.

Consistent with our preregistration (https://osf.io/htf2n), the collected variables and analyses remained unchanged; however, variable labels were adjusted to match the terminology of the more appropriate model by Murphy et al. (23). The nomenclature and definitions of interoceptive measures and facets vary across the literature; therefore, we provide below an overview of the measures and facets included in this study based on the model by Murphy et al. (23).

**Measurement (experiment)**	**What is measured?**	**How is it measured?**
**d'** (HDT)	Interoceptive accuracy	Behavioral (objective)
**Confidence ratings** (HDT)	Interoceptive accuracy	Self-reported (subjective)
**MAIA-2 scores** (Questionnaire)	Interoceptive attention	Self-reported (subjective)
**Intensity ratings** (VIA task)	Interoceptive attention	Self-reported (subjective)
**Neural responses** (VIA task)	Interoceptive attention	Physiological (objective)
**meta-d'** (HDT)	Metacognitive interoceptive awareness (sensitivity)	Combines objective and subjective measures
**meta-d'/d' ratio** (HDT)	Metacognitive interoceptive awareness (efficiency)	Combines objective and subjective measures

Note. HDT, heartbeat discrimination task; MAIA-2, Multidimensional Assessment of Interoceptive Awareness Version 2*; VIA task, visceral interoceptive awareness task*

The interoceptive facets investigated in this study are defined as follows*:*

Interoceptive accuracy: the correspondence of perceived and actual body signals

Interoceptive attention: the degree to which interoception is the object of attention

Metacognitive interoceptive awareness*:* the metacognitive evaluation of experience/performance such as confidence-accuracy correspondence, referred to as interoceptive insight in Khalsa et al. (24)

- *Metacognitive sensitivity:* how well a person can recognize the accuracy or inaccuracy of their judgments
- *Metacognitive efficiency:* metacognitive sensitivity relative to task performance

Please note that definitions of the term interoceptive awareness can vary across the literature, but it is usually defined to *"encompass any (or all) of the different interoception features accessible to conscious self-report"* (24, p.502). Within the model framework proposed by Murphy and colleagues (23; p.1), interoceptive awareness is conceptualized as a metacognitive measure reflecting *"the correspondence between interoceptive accuracy and interoceptive sensibility",* referred to as interoceptive insight by Khalsa and colleagues (24). In the main manuscript, we refer to this measure as metacognitive awareness. In this context, the names of the MAIA-2 and the VIA task may be misleading, as they include the term "awareness," which does not correspond to our definition of metacognitive awareness. Instead, the questionnaire and task ratings are conceptualized as measures of attention.

As part of a larger study, additional variables were assessed before, after, and during residential treatment (reported elsewhere).

Supplementary Results

Questionnaire data

To determine whether the significant correlation between self-reported interoceptive attention (MAIA-2 scores) and interpersonal problems (IIP scores) was driven by an outlier in the patient group, we recalculated the correlation excluding that subject. The correlation remained significant within BPD patients ($r_{(50)}$=-0.42, $p_{(cor)}$=0.004) and across both groups ($r_{(50)}$=-0.62, $p_{(cor)}$<0.0001).

Heartbeat discrimination task

Before the analysis, all trials from the heartbeat discrimination task were plotted and screened for noise. Noisy trials were identified and excluded from further analysis. If the number of noisy trials exceeded 20% of all trials (i.e., more than 16 trials), the entire subject was excluded from further analysis. This procedure resulted in the exclusion of 9 BPD patients and 0 HC from the baseline analysis and an additional 4 BPD patients and 3 HC from the analysis of DBT effects.

The mixed-design ANCOVA with group as between-subject factor (HC, BPD) and trial type (correct, incorrect) as within-subject factor revealed no significant group effect nor an interaction effect with group on confidence ratings of the heartbeat discrimination task (all *p*-values > 0.05). Additionally, there were no treatment-related effects on confidence ratings within BPD patients and compared to HC (all *p*-values > 0.05).

The d'-values in the HC group clustered around zero, suggesting that the task was very challenging. However, we tested whether d'-values of the HC group were significantly different from zero using a one-sample t-test. Mean d'-values of HC (0.29±0.41) were significantly higher than zero ($t_{(25)}$= 3.68, *p*=0.001), indicating above-chance performance. Similarly, BPD patients also showed mean d'-values (0.20±0.47) that were significantly different from zero ($t_{(39)}$= 2.77, *p*=0.009).

Some BPD patients exhibited negative d'-values, indicating performance below chance level. To account for the potential influence of these values on the moderation effect of symptom severity (BSL-23 scores) on behavioral interoceptive accuracy, we set all negative d'-values to zero and repeated the moderation analysis. The results showed that the moderation effect of BSL-23 scores × group on behavioral interoceptive accuracy remained significant ($F_{(1,58)}$=10.50, *p*=0.002, η_p^2=0.15).

Mediation analysis

A mediation analysis using childhood maltreatment (CTQ scores) as a predictor, self-reported interoceptive attention as a mediator (MAIA-2 scores), and interpersonal problems (IIP scores) as the outcome measure further revealed a significant direct effect of childhood maltreatment on interpersonal problems (B=0.01, $p < 0.0001$) but no significant mediation effect (B=0.001, p=0.43) of self-reported interoceptive attention on this relation.

Intensity ratings of the VIA task

The mixed-design ANCOVA with group as between-subject factor (HC, BPD) and condition (stomach, heart, target) as within-subject factor revealed no significant group effect nor an interaction effect with group (all p-values > 0.05) but a significant main effect of condition ($F_{(2,156)}$=7.64, p<0.01, η_G^2=0.06) on the intensity ratings of the VIA task. As expected, the exteroceptive condition target (HC: 6.04 ± 1.74, BPD: 6.19 ± 1.68) was rated as more intense than the interoceptive conditions stomach (HC: 5.65 ± 1.50, BPD: 4.47 ± 2.19) and heart (HC: 5.29 ± 1.71, BPD: 5.05 ± 1.79) in both groups (stomach vs. target: $t_{(79)}$=-4.38, $p_{(cor)}$=0.0001; heart vs. target: $t_{(79)}$=-3.36, $p_{(cor)}$=0.002).

Exploratory whole brain level analysis

An exploratory whole brain level analysis revealed group differences in the left supplementary motor area for interoceptive attention to the stomach relative to exteroceptive attention (MNI: -10, 4, 56; $t_{(72)}$=4.84; p_{FWE}=0.011) prior to treatment. Further, activity of the right inferior frontal gyrus, triangular part changed significantly after treatment within BPD patients for attention to the heart compared to exteroception (MNI: 36, 28, 28; $t_{(26)}$=7.71; p_{FWE}=0.046). There were multiple changes in neural activity of different brain regions in BPD patients compared to HC after treatment (see **Supplementary Tab. 4**). We further detected a significant treatment response × time interaction effect in the right precuneus (MNI: 6, -46, 58; $t_{(25)}$=5.10; p_{FWE}=0.004) for interoceptive attention versus exteroceptive attention after treatment within BPD patients. Significance was assessed at cluster level with $p < 0.05$, FWE corrected and brain regions were identified with the Automated Anatomical Labeling (AAL) Atlas (25, 26).

RSA bootstrapping

As exact bootstrapped p-values vary slightly across iterations, we report multiple comparison–corrected bootstrapped p-values ($p_{(corBoot)}$) from a representative iteration. These values reflect

comparisons of neural representations during cardiac interoceptive attention versus exteroceptive attention in BPD patients compared to HC. Specifically, BPD patients exhibited greater similarity in neural activity patterns between interoceptive attention to the heart and the exteroceptive condition, characterized by significantly reduced negative correlations compared to HC in both regions (insular cortex: BPD: $r_{(48)}$=-0.51, HC: $r_{(29)}$=-0.61, $t_{(79)}$=3.18, $p_{(corBoot)}$=0.007, d=0.69; dACC: BPD: $r_{(48)}$=-0.49, HC: $r_{(29)}$=-0.61, $t_{(79)}$=2.46, $p_{(corBoot)}$=0.046, d=0.56).

RSA results control analysis

As preregistered, bootstrapped t-tests were used to examine differences in neural representations of interoceptive attention between BPD patients and HC. To assess whether the observed group differences in neural activation within the insular cortex and the dACC remained significant after controlling for potential confounding variables (age, sex, BMI, psychotropic medication use, hunger, the urge to urinate, and prescan inner tension), we conducted additional ANCOVAs. These models included group (HC, BPD) and the covariates as predictors, with the z-transformed correlation coefficients of the RSAs as the dependent variable. Results from both ANCOVAs indicated that the group differences remained statistically significant after adjusting for covariates (main effect of group insular cortex: $F_{(1,72)}$=7.95, p=0.006, η_p^2=0.10; dACC: $F_{(1,72)}$=6.25, p=0.02, η_p^2=0.08).

Additional control analyses

We calculated whether there were differences in baseline symptom severity between the BPD patients who did not complete the post-measurements (regardless of the reason for discontinuation, n=18) and those who completed all assessments (n=37). We found no significant differences in symptom severity ($t_{(53)}$ = -1.47, p = 0.15) between the two groups. Descriptive differences were observed in psychotropic medication use and comorbidities. Approximately 81% of BPD patients who completed all assessments were receiving psychotropic medication, compared to 50% of those who completed only the pre-measurement. The mean number of psychotropic medications was higher in the completer group (1.75 vs. 0.77). In addition, a greater proportion of completers reported current (94% vs. 75%) and lifetime (83% vs. 56%) comorbid disorders.

Supplementary Tab. 5 reports Bayes factors for models indicating no significant effects of group or time across the following outcomes: behavioral interoceptive accuracy (d'), metacognitive sensitivity and efficiency (meta-d' and meta-d'/d' ratio), self-reported interoceptive accuracy (average confidence ratings), self-reported interoceptive attention (intensity ratings averaged across interoceptive

conditions), and physiological interoceptive attention (RSA coefficients for all non-significant comparisons).

Additional exploratory analyses

The z-transformed correlation coefficients of the RSAs were not significantly associated with the intensity ratings of the VIA task, averaged across interoceptive conditions (all p-values > 0.05).

There was a trend towards a significant moderation effect of group × BSL-23 scores on confidence ratings, averaged across correct and incorrect trials of the heartbeat discrimination task ($F_{(1,58)}$=3.71, p=0.059).

Supplementary Discussion

Exploratory whole brain level results

We discuss the exploratory whole-brain findings in the following. Due to their exploratory nature and the lack of specific hypotheses, these results are preliminary, should be interpreted cautiously, and warrant replication.

Alterations in the supplementary motor area (SMA) in BPD patients compared to HC during attention to the stomach further support altered interoceptive attention in BPD. The SMA has previously been shown to be engaged during interoceptive attention tasks (27, 28). Interestingly, functional connectivity to this region has been associated with altered gut-brain communication in functional dyspepsia (29), suggesting that SMA involvement may reflect differences in the processing of visceral signals in BPD patients compared to HC.

Changes in inferior frontal gyrus (IFG) activity during attention to the heart after treatment in BPD patients may be explained by several factors. First, in his influential work on interoceptive awareness, Craig (30) noted that activation in the anterior insular cortex (AIC) often extends into the frontal operculum and adjacent inferior frontal regions, and he therefore frequently refers to AIC/IFG activity when describing neural correlates of interoceptive processing. The observed IFG activation therefore further supports our findings of altered neural responses during cardiac interoception in BPD patients. Second, BPD has frequently been associated with amygdala hyperactivity to affective stimuli, which has been linked to impaired prefrontal control (31, 32). Previous research suggests that fronto-insular responses are associated with positive treatment outcomes following cognitive behavioral therapy (33). Moreover, studies report that after DBT, activity in the inferior frontal gyrus decreases in response to emotional stimuli but increases during tasks requiring inhibitory control (34). Taken together, the present findings may therefore also reflect treatment-related improvements in emotion regulation and cognitive control in BPD patients.

The precuneus is considered part of the posterior default mode network (pDMN) and has been linked to self-awareness, self-referential processes, and internally directed attention (35, 36). Interestingly, a recent study reported greater precuneus activity in healthy participants with lower subjective mindfulness (37). However, Craig (30) noted that although the precuneus is involved in self-referential episodic memory retrieval, its activity tends to show an inverse relationship with activation in the AIC, a region linked to awareness and task-focused attention. Changes in precuneus activity during interoceptive attention compared to exteroceptive attention in treatment responders compared to non-responders may therefore reflect alterations in self-referential processing, potentially related to the focus on mindfulness and awareness during DBT.

Regarding treatment-related changes in BPD patients compared to HC (**Supplementary Tab. 4**), we observed several differences in visual brain regions, including the calcarine fissure, superior occipital gyrus, and lingual gyrus. These effects may be related to the visual component of the interoceptive task, as cues for all conditions were presented as words on a screen during the entire duration of the conditions. Given that BPD patients have been reported to show heightened vigilance toward emotionally salient stimuli (32, 31), these findings may reflect increased reactivity to the visual cues associated with interoceptive conditions, which may carry greater emotional salience and which possibly improved after treatment in BPD patients. Interestingly, a study investigating the effects of mindfulness training on depressive rumination found that mindfulness-based cognitive therapy was associated with reduced connectivity between the salience network and the lingual gyrus during ruminative states, and that this change was linked to improvements in the ability to maintain and regulate attention toward bodily sensations (38).

As discussed above, activation changes in the middle frontal gyrus in BPD patients compared to HC during interoceptive compared to exteroceptive attention may similarly reflect improvements in emotion regulation or cognitive control following treatment.

We also observed additional changes in thalamic activation during interoceptive attention to the heart in BPD patients compared to HC after treatment. The thalamus plays a central role in relaying sensory and interoceptive signals, transmitting afferent information from peripheral interoceptors to cortical regions (24, 39, 40). Supporting this role, recent work has demonstrated that single neurons in thalamic and subthalamic regions process cardiac and respiratory signals in humans (40). Together, these findings may indicate treatment-related changes in the processing and integration of interoceptive signals in BPD patients compared to HC, particularly for cardiac signals.

Supplementary Tables

Supplementary Table S1: Sample description for patients with BPD and HC. The higher number of women than men reflects the distribution of BPD patients treated in clinical settings (41). All study participants are Caucasian.

	BPD patients (n=55)		HC (n=31)		
Characteristics	M ± SD or *n*	Min/Max	M ± SD or *n*	Min/Max	*X^2 / t-test, p*
Demographic data					
Age, years	28.3 ± 10.2	18/57	28.0 ± 10.9	18/60	0.9
Sex, male/female	7/48		4/27		1
Gender, male/female/diverse	6/47/2		4/27/0		
BMI	29.6 ± 7.1	16.2/45	24.2 ± 4.5	18.8/36.6	<0.0001
Education, years	13.6 ± 2.8*	9/24	17.3 ± 3.0	13/24	<0.0001
Psychological data					
Depression (BDI-II)	37.2 ± 12.5	6/57	3.6 ± 3.7	0/15	<0.0001
Social anxiety (LSAS)	76.3 ± 30.6	1/124	24.5 ± 11.5	4/44	<0.0001

*Notes. BDI-II, Beck Depression Inventory-II; BMI, Body Mass Index; BPD, borderline personality disorder; HC, healthy controls; LSAS, Liebowitz Social Anxiety Scale; M, Mean; n, number of available data points; SD, Standard Deviation; * data not available for 5 BPD patients*

Supplementary Table S2: Axis I disorders and psychotropic medication of BPD patients and HC.

	BPD patients	HC
Characteristics	*n*	*n*
Axis I disorders (DSM-V criteria)		
Any current comorbid diagnosis, yes/no	48/7	0/31
Any lifetime comorbid diagnosis, yes/no	53/2	0/31
Psychotropic drugs		
Antidepressants	35	0
Antipsychotics	22	0
Psychostimulants	6	0
Anticonvulsants	2	0

Notes. BPD, borderline personality disorder; HC, healthy controls; n, number of available data points

Supplementary Table S3: Longitudinal sample description for patients with BPD and HC.

	BPD patients (n=37)		HC (n=31)		
Characteristics	M ± SD or *n*	Min/Max	M ± SD or *n*	Min/Max	X^2 / *t-test, p*
Demographic data					
Age, years	28.1 ± 10.7	19/57	28.0 ± 10.9	18/60	1
Sex, male/female	5/32		4/27		1
Gender, male/female/diverse	4/32/1		4/27/0		
BMI	29.7 ± 6.6	16.2/45	24.2 ± 4.5	18.8/36.6	<0.001
Education, years	14.2 ± 2.7*	10/24	17.3 ± 3.0	13/24	<0.001
Psychological data					
Depression (BDI-II)	40.0 ± 9.4	6/57	3.6 ± 3.7	0/15	<0.0001
Social anxiety (LSAS)	79.9 ± 29.6	1/124	24.5 ± 11.5	4/44	<0.0001

*Notes. BMI, Body Mass Index; BPD, borderline personality disorder; HC, healthy controls; M, Mean; n, number of available data points; SD, Standard Deviation; * data not available for 2 BPD patients*

Supplementary Table S4: Whole brain results for treatment-related changes of BPD patients compared to HC.

1st level contrast	2nd level contrast	MNI coordinates	*t*-score	p_{FWE}	Brain region
Pre $_{interoception > target}$ > *Post* $_{interoception > target}$	BPD > HC	-38 8 52	5.24	0.011	Left middle frontal gyrus
		16 -72 16	4.61	>0.0001	Right calcarine fissure and surrounding cortex
		0 -80 -8	4.43	>0.0001	Left calcarine fissure and surrounding cortex
		22 -92 24	4.10	0.026	Right superior occipital gyrus
		-10 -74 26	4.01	0.034	Left cuneus
Pre $_{stomach > target}$ > *Post* $_{stomach > target}$	BPD > HC	18 -68 8	4.84	0.001	Right calcarine fissure and surrounding cortex
		-16 76 -12	4.17	0.002	Left lingual gyrus
Pre $_{heart > target}$ > *Post* $_{heart > target}$	BPD > HC	-40 8 52	5.14	0.004	Left middle frontal gyrus
		8 -28 4	5.11	0.006	Right thalamus
		-6 -16 6	5.10	0.009	Left thalamus
		0 -80 -8	4.59	>0.0001	Left lingual gyrus
		-24 -78 -14	4.28	0.003	Left lingual gyrus

Notes. BPD, borderline personality disorder; HC, healthy controls; MNI, Montreal Neurological Institute; FWE, family-wise error corrected

Supplementary Table S5: Bayes factors for models revealing no significant group or time differences across the following outcomes:

	Cross-sectional	*Longitudinal*
Model outcome	*BF_{10}*	*BF_{10}*
behavioral interoceptive accuracy (d')	0.005	0.700
metacognitive sensitivity (meta-d')	0.027	0.123
metacognitive efficiency (meta-d'/d' ratio)	0.013	0.140
self-reported interoceptive accuracy (average confidence ratings)	0.234	0.247
self-reported interoceptive attention (interoceptive intensity ratings)	0.190	0.019
Physiological interoceptive attention (RSA coFefficients)		
IC heart x target	-	0.749
IC stomach x target	0.245	0.293
dACC heart x target	-	0.443
dACC stomach x target	0.237	0.275
Amyg heart x target	1.332	0.192
Amyg stomach x target	0.278	0.204
vmPFC heart x target	0.457	0.354
vmPFC stomach x target	0.237	0.965

Notes. BF_{10}, Bayes factor (evidence for H_1 relative to H_0); IC, insular cortex; dACC, dorsal anterior cingulate cortex; Amyg, amygdala; vmPFC, ventromedial prefrontal cortex

References

1. Faul F, Erdfelder E, Lang A-G, Buchner A. G*Power 3: a flexible statistical power analysis program for the social, behavioral, and biomedical sciences. Behav Res Methods 2007; 39(2):175–91.
2. Storebø OJ, Stoffers-Winterling JM, Völlm BA, Kongerslev MT, Mattivi JT, Jørgensen MS et al. Psychological therapies for people with borderline personality disorder. Cochrane Database Syst Rev 2020; May 4;5(5):CD012955.
3. Bohus M, Haaf B, Simms T, Limberger MF, Schmahl C, Unckel C et al. Effectiveness of inpatient dialectical behavioral therapy for borderline personality disorder: a controlled trial. Behav Res Ther 2004; 42(5):487–99.
4. Liebke L, Bungert M, Thome J, Hauschild S, Gescher DM, Schmahl C et al. Loneliness, social networks, and social functioning in borderline personality disorder. Personal Disord 2017; 8(4):349–56.
5. Bohus M, Wolf-Arehult M. Interaktives Skillstraining für Borderline-Patienten: das Therapeutenmanual. 2nd ed. Schattauer Verlag (Klett); 2018. Available from: URL: https://books.google.de/books?id=TGDlIT8RJQ8C.
6. Linehan MM. DBT skills training manual. Second edition. New York, London: The Guilford Press; 2015.
7. First MB, Williams JBW, Karg RS, Spitzer RL. Structured clinical interview for DSM-5 disorders: SCID-5-CV clinician version. Arlington, VA: American Psychiatric Association Publishing; 2016.
8. Zanarini MC, Vujanovic AA, Parachini EA, Boulanger JL, Frankenburg FR, Hennen J. Zanarini Rating Scale for Borderline Personality Disorder (ZAN-BPD): a continuous measure of DSM-IV borderline psychopathology. J Pers Disord 2003; 17(3):233–42.
9. Doering S, Renn D, Höfer S, Rumpold G, Smrekar U, Janecke N et al. Validierung der deutschen Version des Fragebogens zur Erfassung von DSM-IV Persönlichkeitsstorungen (ADP-IV). Z Psychosom Med Psychother 2007; 53(2):111–28.
10. Beck AT, Steer RA, Brown GK. Manual for the beck depression inventory-II. San Antonio TX: Psychological Corporation; 1996.
11. Liebowitz MR. Social phobia. Mod Probl Pharmacopsychiatry 1987; 22:141–73.
12. Brener J, Liu X, Ring C. A method of constant stimuli for examining heartbeat detection: comparison with the Brener-Kluvitse and Whitehead methods. Psychophysiology 1993; 30(6):657–65.
13. Garfinkel SN, Seth AK, Barrett AB, Suzuki K, Critchley HD. Knowing your own heart: distinguishing interoceptive accuracy from interoceptive awareness. Biol Psychol 2015; 104:65–74.
14. R Core Team. R: A Language and Environment for Statistical Computing. Vienna, Austria; 2021. Available from: URL: https://www.R-project.org/.
15. Cohen J. Statistical Power Analysis for the Behavioral Sciences. New York, NY: Routledge Academic; 1988.
16. Olejnik S, Algina J. Generalized eta and omega squared statistics: measures of effect size for some common research designs. Psychol Methods 2003; 8(4):434–47.
17. Esteban O, Markiewicz CJ, Blair RW, Moodie CA, Isik AI, Erramuzpe A et al. fMRIPrep: a robust preprocessing pipeline for functional MRI. Nat Methods 2019; 16(1):111–6.

18. Esteban O, Markiewicz CJ, Burns C, Goncalves M, Jarecka D, Ziegler E et al. nipy/nipype: 1.8.3: Zenodo; 2022.

19. Gorgolewski K, Burns CD, Madison C, Clark D, Halchenko YO, Waskom ML et al. Nipype: a flexible, lightweight and extensible neuroimaging data processing framework in python. Front Neuroinform 2011; 5:13.

20. Fan L, Li H, Zhuo J, Zhang Y, Wang J, Chen L et al. The Human Brainnetome Atlas: A New Brain Atlas Based on Connectional Architecture. Cereb Cortex 2016; 26(8):3508–26.

21. Xu K, Liu Y, Zhan Y, Ren J, Jiang T. BRANT: A Versatile and Extendable Resting-State fMRI Toolkit. Front Neuroinform 2018; 12:52.

22. Hebart MN, Görgen K, Haynes J-D. The Decoding Toolbox (TDT): a versatile software package for multivariate analyses of functional imaging data. Front Neuroinform 2014; 8:88.

23. Murphy J, Catmur C, Bird G. Classifying individual differences in interoception: Implications for the measurement of interoceptive awareness. Psychon Bull Rev 2019; 26(5):1467–71.

24. Khalsa SS, Adolphs R, Cameron OG, Critchley HD, Davenport PW, Feinstein JS et al. Interoception and Mental Health: A Roadmap. Biol Psychiatry Cogn Neurosci Neuroimaging 2018; 3(6):501–13.

25. Rolls ET, Huang C-C, Lin C-P, Feng J, Joliot M. Automated anatomical labelling atlas 3. NeuroImage 2020; 206:116189.

26. Tzourio-Mazoyer N, Landeau B, Papathanassiou D, Crivello F, Etard O, Delcroix N et al. Automated anatomical labeling of activations in SPM using a macroscopic anatomical parcellation of the MNI MRI single-subject brain. NeuroImage 2002; 15(1):273–89.

27. Haruki Y, Ogawa K. Role of anatomical insular subdivisions in interoception: Interoceptive attention and accuracy have dissociable substrates. Eur J Neurosci 2021; 53(8):2669–80.

28. Critchley HD, Wiens S, Rotshtein P, Ohman A, Dolan RJ. Neural systems supporting interoceptive awareness. Nat Neurosci 2004; 7(2):189–95.

29. Sclocco R, Fisher H, Staley R, Han K, Mendez A, Bolender A et al. Cine gastric MRI reveals altered Gut-Brain Axis in Functional Dyspepsia: gastric motility is linked with brainstem-cortical fMRI connectivity. Neurogastroenterol Motil 2022; 34(10):e14396.

30. Craig AD. How do you feel - now? The anterior insula and human awareness. Nat Rev Neurosci 2009; 10(1):59–70.

31. Degasperi G, Cristea IA, Di Rosa E, Costa C, Gentili C. Parsing variability in borderline personality disorder: a meta-analysis of neuroimaging studies. Transl Psychiatry 2021; 11(1):314.

32. Schulze L, Schulze A, Renneberg B, Schmahl C, Niedtfeld I. Neural Correlates of Affective Disturbances: A Comparative Meta-analysis of Negative Affect Processing in Borderline Personality Disorder, Major Depressive Disorder, and Posttraumatic Stress Disorder. Biol Psychiatry Cogn Neurosci Neuroimaging 2019; 4(3):220–32.

33. Picó-Pérez M, Fullana MA, Albajes-Eizagirre A, Vega D, Marco-Pallarés J, Vilar A et al. Neural predictors of cognitive-behavior therapy outcome in anxiety-related disorders: a meta-analysis of task-based fMRI studies. Psychol Med 2023; 53(8):3387–95.

34. Iskric A, Barkley-Levenson E. Neural Changes in Borderline Personality Disorder After Dialectical Behavior Therapy-A Review. Front. Psychiatry 2021; 12:772081.

35. Zhang R, Volkow ND. Brain default-mode network dysfunction in addiction. NeuroImage 2019; 200:313–31.

36. Menon V. 20 years of the default mode network: A review and synthesis. Neuron 2023; 111(16):2469–87.

37. Ramanathan D, Nan J, Grennan G, Jaiswal S, Purpura S, Manchanda J et al. Modulation of Posterior Default Mode Network Activity During Interoceptive Attention and Relation to Mindfulness. Biol Psychiatry Glob Open Sci. 2024; 4(6):100384.

38. van der Velden AM, Scholl J, Elmholdt E-M, Fjorback LO, Harmer CJ, Lazar SW et al. Mindfulness Training Changes Brain Dynamics During Depressive Rumination: A Randomized Controlled Trial. Biol Psychiatry 2023; 93(3):233–42.

39. Chen WG, Schloesser D, Arensdorf AM, Simmons JM, Cui C, Valentino R et al. The Emerging Science of Interoception: Sensing, Integrating, Interpreting, and Regulating Signals within the Self. Trends Neurosci 2021; 44(1):3–16.

40. Falco E de, Solcà M, Bernasconi F, Babo-Rebelo M, Young N, Sammartino F et al. Single neurons in the thalamus and subthalamic nucleus process cardiac and respiratory signals in humans. Proc Natl Acad Sci U S A 2024; 121(11):e2316365121.

41. Leichsenring F, Heim N, Leweke F, Spitzer C, Steinert C, Kernberg OF. Borderline Personality Disorder: A Review. JAMA 2023; 329(8):670–9.
